# Stay with me: rPL4 kidnaps the viral replicase to limit TVBMV infection

**DOI:** 10.1093/plphys/kiag078

**Published:** 2026-02-25

**Authors:** Mireia Uranga

**Affiliations:** Assistant Features Editor, Plant Physiology, American Society of Plant Biologists, Rockville, MD 20855-2768, USA; Institute for Integrative Systems Biology (I2SysBio), Universitat de València-CSIC, 46908 Paterna, Valencia, Spain

Viruses are intracellular parasites that entirely rely on the host cell to fulfill their life cycle. Due to the limited coding capacity of their genome, positive-sense RNA viruses have evolved strategies to hijack cellular components and pathways for their own purposes (Pollari et al 2024). Many host proteins are recruited by viral proteins to play active roles and favor virus propagation, but killing the host is not favorable for the parasitic nature of the virus. Thus, cell-intrinsic restriction factors act as negative regulators at specific phases to control infection (Garcia-Ruiz 2019). Although these restriction factors represent a promising strategy for breeding virus-resistant crops, their identities and molecular functionality remain poorly understood.

Ribosomes are the plant cell's core translational machinery responsible for producing cellular and organelle-specific proteins (Martinez-Seidel et al 2020). Beyond their essential function in ribosomal subunit assembly and protein synthesis, ribosomal proteins (RPs) are key players in plant development, hormone signaling, and diverse environmental responses (Romani et al 2012; Ramos et al 2020). Moreover, increasing evidence suggests that RPs differentially modulate plant virus infections by taking a proviral or antiviral role (Hafrén et al 2013; Rajamäki et al 2017; Cheng et al 2021), but many questions remain unanswered regarding the mechanisms of action.

In a previous study, Cheng et al (2021) identified the *Nicotiana benthamiana* chloroplastic large ribosomal protein 1 (NbRLP1) as a potential factor promoting infection by tobacco vein banding mosaic virus (TVBMV, genus *Potyvirus*). NbRPL1 interacts with the viral RNA-dependent RNA polymerase nuclear inclusion b (NIb) in the chloroplast, thus reducing its degradation and boosting TVBMV infection.

Recently in *Plant Physiology*, the authors followed up the study of large ribosomal proteins by characterizing the role of the nonchloroplastic NbRLP4 in TBVMV infection, specifically through its interaction with NIb (Chen et al 2026). Subcellular studies revealed that RPL4 localizes to the host cell nucleus with a minor presence in the cytoplasm, showing a very similar distribution to that of NIb. Since co-localization is essential for the interaction between 2 proteins, the authors combined in vitro and in planta approaches to validate the biological interplay between NbRPL4 and NIb. They then segmented and truncated the coding sequence of NbRPL4 to identify the key amino acid residues involved in this interaction. The C3 region of NbRPL4 was identified as crucial for this role, and an alanine replacement assay revealed that 5 specific residues at positions 389 to 393 were responsible for the NIb–NbRPL4 interaction.

To further study the biological function of NbRPL4 in virus infection, *N. benthamiana* leaves were inoculated with a TVBMV vector expressing either wild-type NbRPL4 or its noninteracting mutant (NbRPL4 389 to 393^5A^). At 5 d postinoculation, plants infected with TVBMV-NbRPL4 showed considerably milder vein banding symptoms than those of plants inoculated with TVBMV-NbRPL4 389 to 393^5A^, and the reduction in virus accumulation was confirmed by western blot. These results indicate that transient expression of NbRPL4 inhibited TVBMV replication and subsequent viral infection in plants, an effect that disappeared when the NIb–NbRPL4 interaction was disrupted.

Subsequently, the authors aimed to manipulate the nuclear–cytoplasmic distribution of NbRPL4 by introducing nuclear localization and export signals (NLS and NES, respectively) into its protein coding sequence. Compared to wild-type NbRPL4, plants expressing the NES-NbRPL4 variant exhibited a lower nuclear–cytoplasmic ratio and higher TVBMV accumulation. Conversely, the addition of NLS promoted the nuclear retention of NbRPL4 and reinforced its inhibitory effect on TVBMV replication. Thereby, the antiviral capacity of NbRPL4 seems to be determined by its nuclear localization.

TVBMV replication occurs in the viral replication compartments (VRCs) spread throughout the host cell's cytoplasm. The viral 6K2 protein initiates VRC formation, which recruits viral components (including NIb) and a diversity of host factors to efficiently replicate and translate the viral genome (Zhang et al 2025). Previously, it was reported that exportin 1 (NbXPO1) interacts with NIb from turnip mosaic virus (TuMV) to promote the translocation of the viral protein from the nucleus to the VRCs (Zhang et al 2021). Accordingly, Chen et al (2026) observed that the presence of NbRPL4 hindered the nuclear export of TVBMV NIb, lowering its accumulation in replication vesicles and ultimately impairing TVBMV replication. Conversely, this inhibitory effect on TVBMV infection disappeared when the Nib–NbRPL4 interaction was interrupted. These findings suggest that NbRPL4 interacts with TVBMV NIb to impair the NbXPO1-mediated nuclear export of NIb, leading to a suppression of virus replication (Fig. 1).

**Figure 1:**
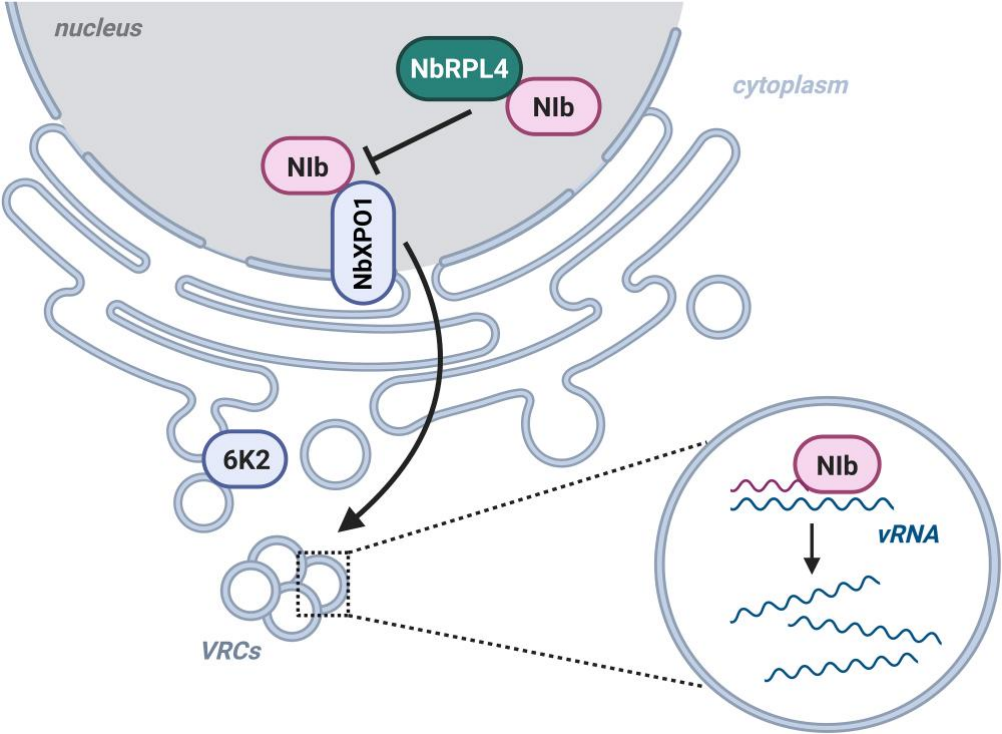
Proposed antiviral role of host ribosomal protein NbRPL4 in tobacco vein banding mosaic virus (TVBMV) replication. The interaction between the viral RNA-dependent RNA polymerase NIb and the host exportin protein NbXPO1 mediates NIb nuclear export. Once in the cytoplasm, NIb gets recruited to the 6K2-induced viral replication complexes (VRCs) to promote viral RNA (vRNA) replication. Nucleus-localized NbRPL4 associates with TVBMV NIb to specifically hinder its NbXPO1-mediated export, which thus inhibits virus replication. Adapted from Figure 9, Chen et al (2026), and created in BioRender.

Regarding the agronomical relevance of potyvirus diseases, the authors also investigated whether NbRPL4 could exert an antiviral effect on potato virus Y (PVY) and TuMV. The NIb proteins of these 2 potyviruses are considerably similar to that of TVBMV (61% and 64% amino acid similarities for PVY and TuMV, respectively) and were also found to interact with NbXPO1. However, the presence of NbRPL4 did not alter the nuclear–cytoplasmic distribution of NIb proteins of PVY or TuMV, nor did it have any inhibitory effect on virus replication.

In summary, Chen et al (2026) demonstrate that NbRPL4 acts as a restriction factor for TVBMV replication by impairing the NbXPO1-mediated nuclear export of NIb in a virus-selective manner. The nuclear–cytoplasmic distribution of NbRPL4 positively correlates with its suppressive effect on TVBMV infection. Further research is needed to identify specific subcellular localization signals and introduce them into NbRPL4, which could promote its nuclear accumulation and reinforce the antiviral effect. Additionally, the characterization of the key amino acid residues of TBVMV NIb involved in the interaction with NbRPL4 could help decipher the antiviral spectrum of this host protein and postulate it as a valuable cell-intrinsic restriction factor against potyvirus diseases. Addressing all these questions will contribute to a better understanding of virus–host interactions, opening new venues for breeding virus-resistant crops.

## Data Availability

None required.
